# Bruceine D and Narclasine inhibit the proliferation of breast cancer cells and the prediction of potential drug targets

**DOI:** 10.1371/journal.pone.0297203

**Published:** 2024-01-12

**Authors:** Xinhao Chen, Hua Li

**Affiliations:** School of Traditional Chinese Medicine, Hunan University of Chinese Medicine, Changsha, China; University of Hawai’i at Manoa, UNITED STATES

## Abstract

**Background:**

Breast cancer is one of the most common female malignancies. This study explored the underlying mechanism through which the two plant compounds (Brucaine D and Narclasine) inhibited the proliferation of breast cancer cells.

**Objective:**

The purpose of this study was to explore the effect of Brucaine D and Narclasine on breast cancer development and their potential drug targets.

**Methods:**

GSE85871 dataset containing 212 samples and the hallmark gene set “h.all.v2023.1.Hs.symbols.gmt” were downloaded from the Gene Expression Omnibus (GEO) database and the Molecular Signatures Database (MSigDB) database, respectively. Principal component analysis (PCA) was applied to classify clusters showing similar gene expression pattern. Single sample gene set enrichment analysis (ssGSEA) was used to calculate the hallmark score for different drug treatment groups. The expressions of genes related to angiogenesis, glycolysis and cell cycle were detected. Protein-protein interaction (PPI) network analysis was performed to study the interaction of the hub genes. Then, HERB database was employed to identify potential target genes for Narclasine and Bruceine D. Finally, *in vitro* experiments were conducted to validate partial drug-target pair.

**Results:**

PCA analysis showed that the significant changes in gene expression patterns took place in 6 drugs treatment groups (Narciclasine, Bruceine D, Japonicone A, 1beta-hydroxyalatolactone, Britanin, and four mixture drugs) in comparison to the remaining drug treatment groups. The ssGSEA pathway enrichment analysis demonstrated that Narciclasine and Bruceine treatments had similar enriched pathways, for instance, suppressed pathways related to angiogenesis, Glycolysis, and cell cycle, etc.. Further gene expression analysis confirmed that Narciclasine and Bruceine had a strong ability to inhibit these cell cycle genes, and that MYC, CHEK2, MELK, CDK4 and EZH2 were closely interacted with each other in the PPI analysis. Drug target prediction revealed that Androgen Receptor (AR) and Estrogen Receptor 1 (ESR1) were the targets for Bruceine D, and Cytochrome P450 3A4 enzyme (CYP3A4) was the target for Narciclasine. Cell experiments also confirmed the connections between Narciclasine and CYP3A4.

**Conclusion:**

The present study uncovered that Narciclasine and Bruceine D could inhibit the growth of breast cancer and also predicted the potential targets for these two drugs, providing a new therapeutic direction for breast cancer patients.

## Introduction

Breast cancer as one of the most common female malignancies with a high heterogeneity [1, 2] accounts for 25–30% of all malignancies among European and American women [3], and its incidence has been increasing in recent years [4]. Moreover, breast cancer is now the most frequent female tumor in developed regions of China [5]. Despite a variety of treatment options, the outcome and survival of breast cancer remains dismal due to its heterogeneous, invasive, recurrent and metastatic nature [4]. A lack of biological targets and comprehensive understanding of the mechanism underlying the tumorigenesis restricts the development of therapeutic strategies for breast cancer [6]. Conventional chemotherapies using taxanes, vinorelbine and anthracyclines are the primary treatments, particularly in preoperative setting [7–9]. In recent years, natural products have been increasingly used as potential anticancer drugs [10]. Bruceine D and Narclasine are two natural products derived from plants that are believed to have anti-cancer ability [11, 12]. CDK4 kinase is an important protein kinase in cell cycle regulation and is closely related to the tumorigenesis and progression of breast cancer. In this study, we explored the effects of Bruceine D and Narclasine on CDK4 and their role in regulating cell cycle in breast cancer.

Cell cycle involves a series of complex events, through which a cell duplicates its contents and divides to produce two genetically identical daughter cells [13]. Cell cycle and its regulation are crucial to cell growth and multiplication and involves regulatory proteins such as cyclin-dependent kinases (CDKs) and cyclin proteins, oncogenes and tumor-suppressor genes [14]. Mitotic checkpoint protein allows cell cycle stages to proceed or to be inhibited [14]. Cell cycle consists of interphases of G1 (beginning of S phase), S (DNA replication) and G2 (beginning of mitosis) phases, and mitotic (M) phase [15]. During the whole cell cycle phase, cyclin proteins and CDKs form complexes and catalyze progression of cell cycle after activation and they are seen as the fundamental aspects of regulation [16]. CDKs as key factors are only activated by cyclins at certain points of cell cycle [17]. When any incomplete process or damage occurs in the cell cycle, cyclin-CDK regulatory activity will be blocked, resulting in arrested cell division cycle until these abnormalities are resolved [18]. Some studies have found that three CDKs (CDK2, CDK4, and CDK6) are involved in interphase regulation, leading to exit from or entry into sub-phases [16]. DNA damage signals inhibit these CDKs, inducing cell-cycle arrest [19]. Activation of CDK4 and CDK6 affects the progression of G1 early stage, then they bind with cyclin-D to phosphorylate the retinoblastoma tumor suppressor protein (RB) responsible for inactivating E2F transcription factors [20]. E2F encoding proteins are necessary for the G1/S transition and facilitates cells to the next cycle phases [21]. CDK4 and CDK6 inhibited by DNA damage detection cannot inactivate RB, which allows RB to bind to E2F and further suppress the activity of E2F, thereby leading to cell cycle arrest [16]. In breast cancer, overactivation of CDK4 causes abnormal cell cycles that promote the proliferation and metastasis of tumor cells [22]. Therefore, inhibition of CDK4 activity has become an important strategy for treating breast cancer.

Bruceine D and Narclasine are two natural products isolated from the shrub *Brucea javanica* (L.) Merr. (Simaroubaceae) [23] and the *Narcissus* species (Amaryllidaceae) [12], respectively. Recent studies have shown that these two compounds have antitumor activity. Bruceine D could inhibit the proliferation and invasion of hepatocellular carcinoma [24] and induce apoptosis and autophagy of lung cancer [25]. Narclasine has anti-proliferative and anti-invasive effects on a variety of cancer cells [12], for example, it inhibits esophageal cancer cell proliferation and migration via suppressing FAK signaling pathway [26]. However, the specific mechanisms through which these two compounds regulate CDK4 signaling and their role in regulating breast cancer cell cycle remained unclear. To bridge such a gap, the present study investigated the effects of the two compounds on CDK4 kinase activity and their regulatory mechanisms in breast cancer cell cycle applying bioinformatics analysis, hoping to contribute to the treatment of breast cancer.

## Material and methods

### Data acquisition

The RNA-sequencing data from 212 human breast cancer cell line (MCF7) samples treated by 102 drugs were downloaded from GEO (GEO: GSE85871, https://www.ncbi.nlm.nih.gov/geo/) database [27].

### Data preprocessing

The expression matrix was read by the GEOquerry R package and the box plot was used to assess differences or abnormal value in data [28]. Then, the mean value in the same group was taken as the gene expression level to perform the Principal Component Analysis (PCA) for dimensionality reduction. Drug-treated groups showing significant changes in expression patterns were extracted from the gene expression data.

### Hallmark enrichment score analysis

The hallmark gene set “h.all.v2023.1.Hs.symbols.gmt” was downloaded from MSigDB (https://www.gsea-msigdb.org/gsea/msigdb/collections.jsp) database [29] and used to compute the hallmark enrichment score of each sample by the single sample gene set enrichment analysis (ssGSEA) algorithm using GSVA R package [30].

### Screening differentially expressed genes (DEGs)

The mean values of gene expression calculated by the t-test were used to indicate the up- or down-regulation in genes from different groups. DEGs were screened under p value < 0.05.

### Protein-protein interaction (PPI) network analysis

The screened hub genes were subjected to PPI networks analysis, and the degree of each gene was calculated by the CytoNCA package in Cytscape software (version 3.8.0), which could be applied to evaluate the connectivity degree of nodes and identify the most important clusters of nodes in a network [31].

### Drug target prediction

HERB database [32] (http://herb.ac.cn/Search/) was used to predict the target for the drugs of Bruceine D and Narclasine. Briefly, we entered the name of Bruceine D and Narclasine in the column of “Ingredient” and clicked the “Search” button for analysis.

### Cell culture and drug treatment

Neoplastic MCF7 and non-neoplastic MCF10A cell lines were purchased from American Type Culture Collection (ATCC, Manassas, VA, USA) and maintained in Dulbecco’s Modified Eagle’s Medium (DMEM, Sigma-Aldrich, USA) supplemented with 10% Fetal Bovine Serum (FBS, Sigma-Aldrich, USA). Narciclasine of ≥ 98% purity was purchased from Biopurify Phytochemical (Chengdu, China). Narciclasine stock solution was prepared applying dimethyl sulphoxide (DMSO) and diluted to final the concentration in culture medium. Accordance to a recently published paper [33], MCF-7 cells were treated with Nar (100 nM) for 24 h.

### Quantitative reverse transcription-polymerase chain reaction (qRT-PCR)

Total mRNA was extracted using the TRIzol reagent (Invitrogen) following the manufacturer’s instruction. QRT-PCR amplifications were conducted adopting the FastStart Essential DNA Green Master and LightCycler 96 Instrument (Roche, Basel, Switzerland). Total mRNA was reverse-transcribed into cDNA using a PrimeScript RT reagent kit (Takara, Shiga, Japan). The PCR was amplified under the following conditions: 50 cycles of 94°C for 10 minutes, 94°C for 10 seconds, and 55°C for 45 seconds. The primer sequences were designed as follows: CYP3A4: forward 5′-GGTGGTGGTGATGATTCC-3′ and reverse 5′-TTGAAGAAGTCCTCCTAAGC-3′; GAPDH: forward 5′- AATGGGCAGCCGTTAGGAAA-3′ and reverse 5′- GCCCAATACGACCAAATCAGAG-3′.

### Statistical analysis

All statistical analysis and visualization were performed using the R software (version 4.3.1). The students test was used to compare the differences between two sets of continuous variables. P value < 0.05 was regarded as statistical difference. Some supporting analyses were provided by Sangerbox (http://sangerbox.com/home.html).

## Results

### Gene expression pattern of MCF7 cell lines after drug treatment

**Fig 1:**
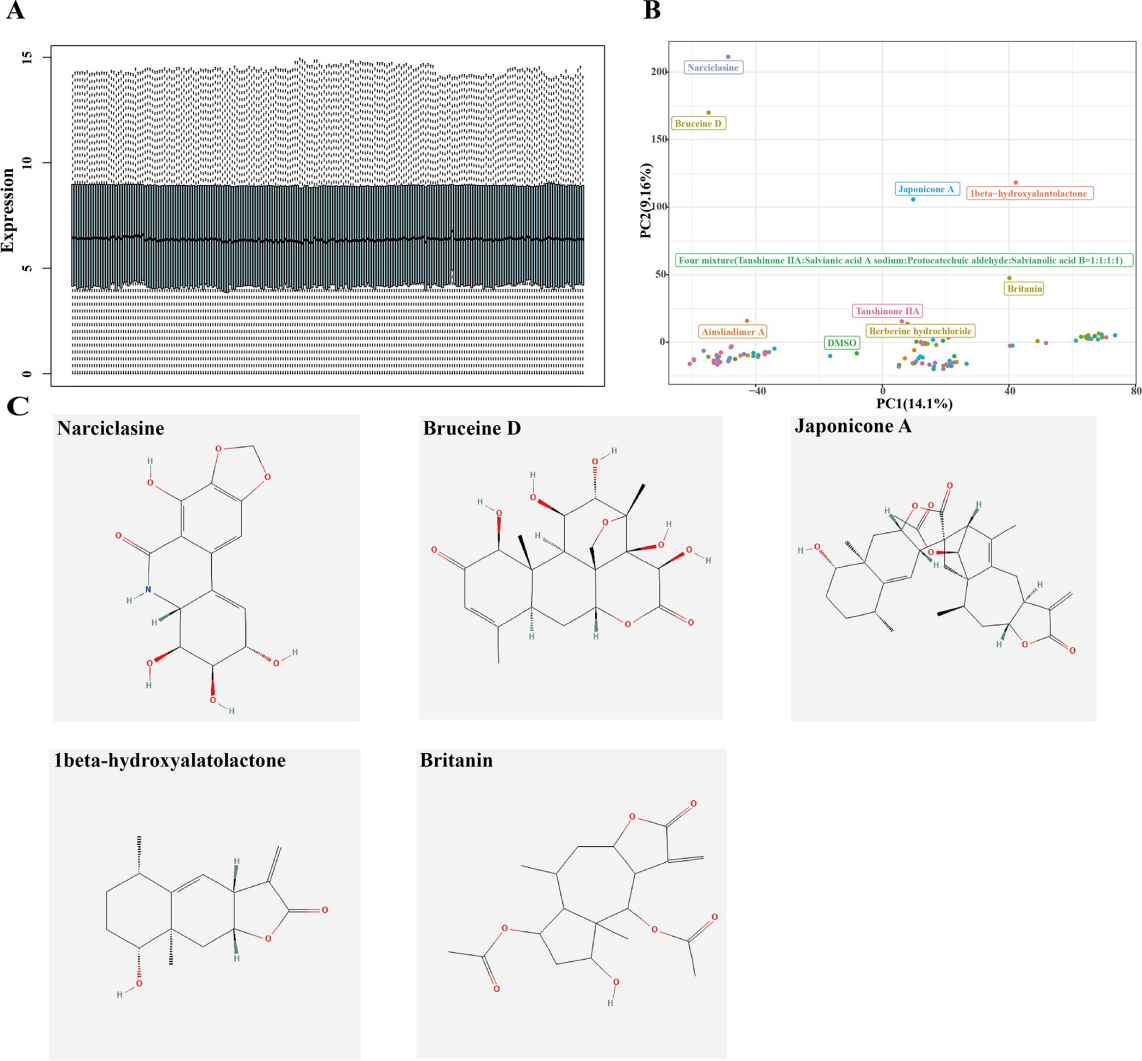
The expression pattern of MCF7 cell line after drugs treatment. (A) The box plot of expression matrix in MCF7 cell lines treated by drugs. (B) PCA analysis of MCF7 cell lines treated by drugs. (C) The 2D structure of the 5 pure chemical compounds.

Visualization of the expression levels of all genes in each sample (**Fig 1A**) showed that the gene expressions were evenly distributed, therefore no normalization was required. PCA analysis revealed that the gene expression pattern had significant changes in the Narciclasine, Bruceine D, Japonicone A, 1beta-hydroxyalatolactone, Britanin and Four-mixture (tanshinone IIA, salvianic acid A sodium, protocatechuic aldehyde, salvianolic acid B) groups compared with other drug treatment groups (**Fig 1B**). The 2D structure of the 5 pure chemical compound drugs were shown in **Fig 1C**. These groups with similar gene expression patterns were named as common groups, and we further explored the differences in pharmacological molecular mechanisms between the significantly changed groups (including six drugs) and common groups.

### Expression profile difference analysis on MCF7 cell lines after drug treatment

**Fig 2:**
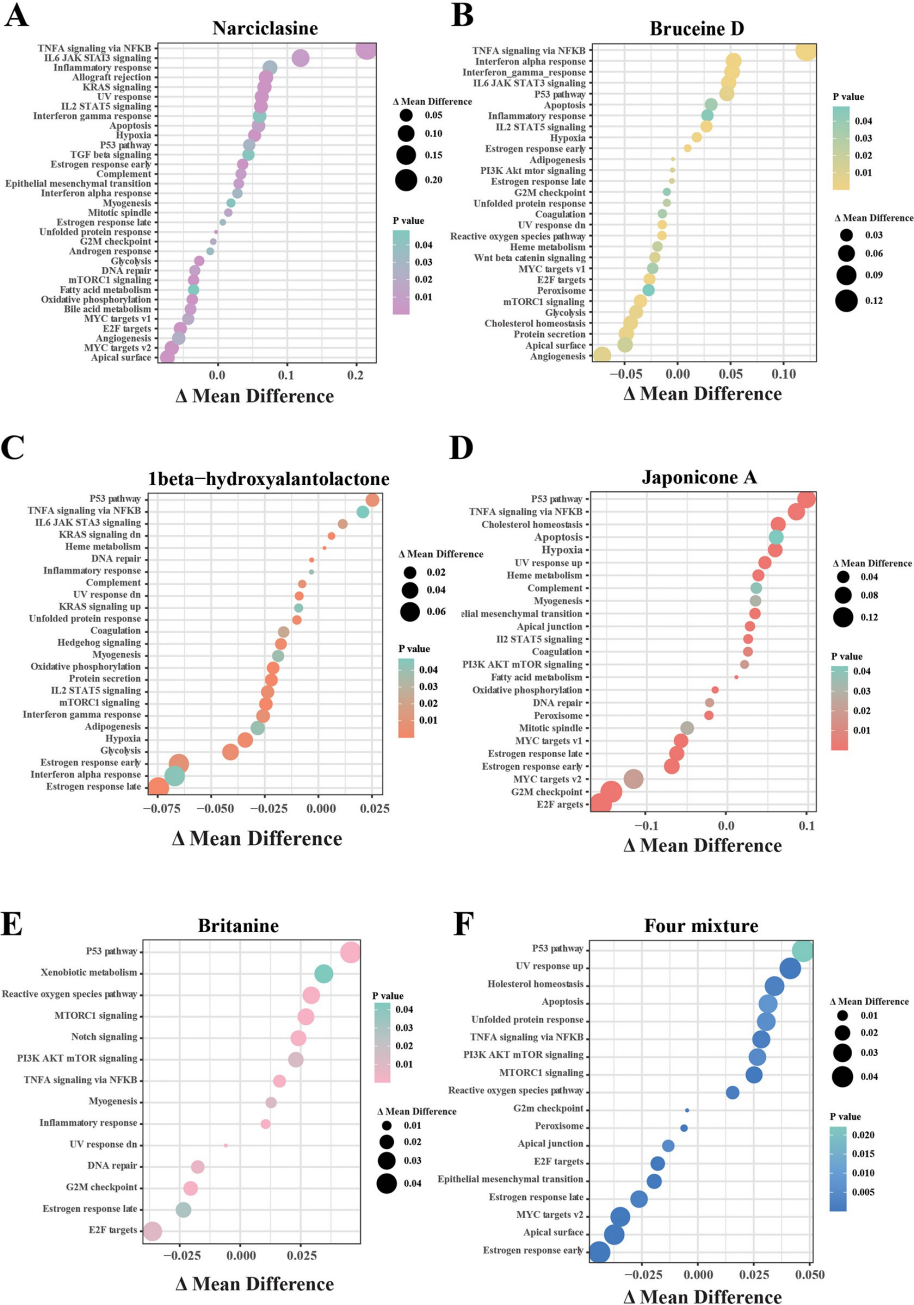
Expression profile feature of MCF7 cell line after drugs treatment. (A) Enrichment scores of MCF7 cells treated by Narciclasine. (B) Enrichment scores of MCF7 cells treated by Bruceine D. (C) Enrichment scores of MCF7 cells treated by Japonicone A. (D) Enrichment scores of MCF7 cells treated by 1beta-hydroxyalatolactone. (E) Enrichment scores of MCF7 cells treated by Britanine. (F) Enrichment scores of MCF7 cells treated by four mixture (tanshinone IIA, salvianic acid A sodium, protocatechuic aldehyde, salvianolic acid B).

To explore the effect of the 6 drugs on the expression profile of MCF7 cell lines, we calculated the ssGSEA score in the corresponding groups in the Hallmark gene set. Specifically, the enrichment pathway in the Narciclasine and Bruceine D treatment groups was similar (**Fig 2A and 2B**), the pathways of Angiogenesis, Glycolysis, G2M checkpoint, Myc targets v1 and E2F targets were suppressed, and the p53, TNFA signaling via NFKB, apoptosis and inflammatory response pathway were activated. The pathways of Glycolysis, Interferon gamma/alpha response in 1beta-hydroxyalatolactone treatment groups were inhibited, while p53 and heme metabolism pathways were activated (**Fig 2C**). Japonicone A suppressed the expression of G2M checkpoint, Myc targets v1, and E2F targets genes (**Fig 2D**). Britanin suppressed the expression of E2F targets and G2M checkpoint genes (**Fig 2E**). Four mixture drugs inhibited the expressions of the target gene for E2F (**Fig 2F**). Combined with the results from PCA clustering and pathway enrichment analysis, we found that tNarciclasine and Bruceine D had similar anti-cancer mechanisms and were distinct from other drug treatment groups. Thus, the pharmacological mechanisms of these two drugs were further studied.

### The angiogenesis and glycolysis were suppressed by Narciclasine and Bruceine D

**Fig 3:**
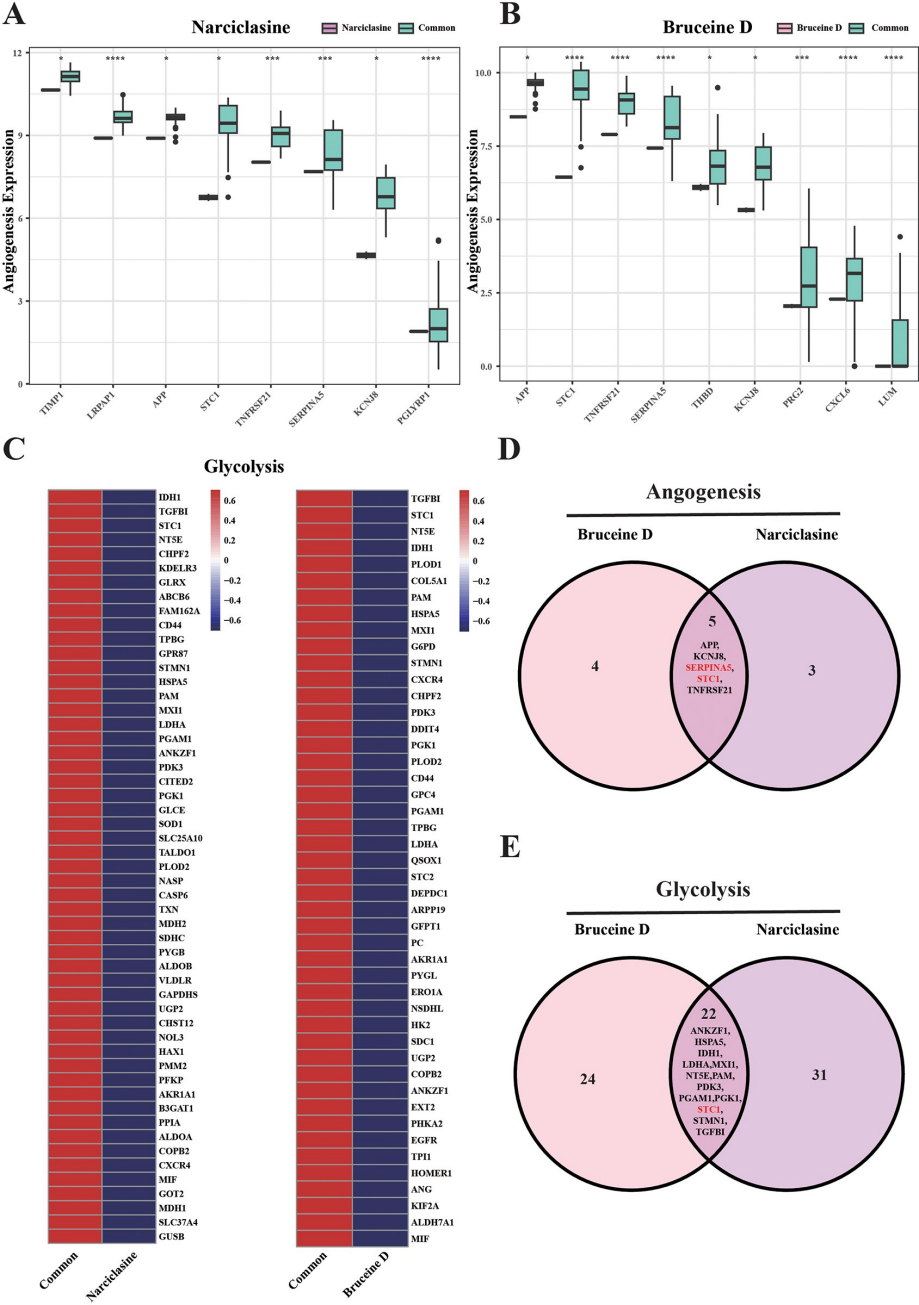
The expression of angiogenesis and glycolysis related genes in comparing with common groups. (A) The expression levels of angiogenesis-related genes in MCF7 cells that treated by Narciclasine. (B) The expression levels of angiogenesis-related genes in MCF7 cells that treated by Bruceine D. (C) The expression levels of glycolysis-related genes in MCF7 cells that treated by Narciclasine and Bruceine D. (D) Venn plot of angiogenesis-related genes in Narciclasine and Bruceine D treatment groups. (E) Venn plot of glycolysis-related genes in Narciclasine and Bruceine D treatment groups. *p < 0.05, ***p < 0.001, ****p < 0.0001. The vertical axis in Fig C and D means the expression level of genes, where the red square represents high expression, and the blue square represents low expression.

Significantly down-regulated DEGs between Narciclasine and Bruceine D treatment groups were screened. As angiogenesis and glycolysis play an important role in tumor development, increase in glycolysis is a major hallmark of tumor progression and can help tumor cells obtain more energy in an oxygen-free environment [34]. Abnormally increased angiogenesis could provide nutrients and oxygen to support cell growth and proliferation of tumors [35] and, in turn, lactic acid produced by glycolysis can promote angiogenesis [36]. Thus, down-regulated DEGs related to angiogenesis and glycolysis were analyzed. Angiogenesis-related DEGs including the APP, KCNJ8, SERNIPA5, STC1 and TNFRSF21 were significantly down-regulated (**Fig 3A and 3B**), and glycolysis-related DEGs including ANKZF1, HSPA5, IDH1, LDHA, MXI1, NT5E, PAM, PDK3, PGAM1, PGK1, STC1, STMN1 and TGFBI were significantly down-regulated (**Fig 3C**). Interestingly, Venn plots of angiogenesis- (**Fig 3D**) and Glycolysis-related genes (**Fig 3E**) showed that the STC1 co-participated in the inhibition of angiogenesis and glycolysis of the two drugs. Previous studies reported that stanniocalcin-1 (SCT1) acted as a tumor growth factor to promote tumor proliferation, and that overexpression of SCT1 can promote tumor proliferation and subcutaneous tumor formation in mice [37], whereas inhibition of SCT1 reduced the tumor cell proliferation [38]. Increasing studies indicated that the effect of STC1 on promoting cell proliferation was closely associated with cell cycle changes. In ovarian cancer, STC1 stimulated high expression of cell cycle-related proteins (cyclin A/B1/D1 and CDK2/4) for rapid proliferation of tumor cells [39] and mitotic cycle of G1 to S was dramatically shortened [40]. In addition, STC1 also activates ERK and JNK pathway promoting inhibitor of apoptosis proteins (BcL-2 and BcL-xl) and inhibits the expression of pro-apoptotic proteins (Bax, Bak and Bid) for tumor survival [41]. These findings suggested that Narciclasine and Bruceine D could inhibit the proliferation of MCF7 cells through regulating cell cycle.

### Narciclasine and Bruceine D inhibited tumor proliferation through regulating cell cycle

**Fig 4:**
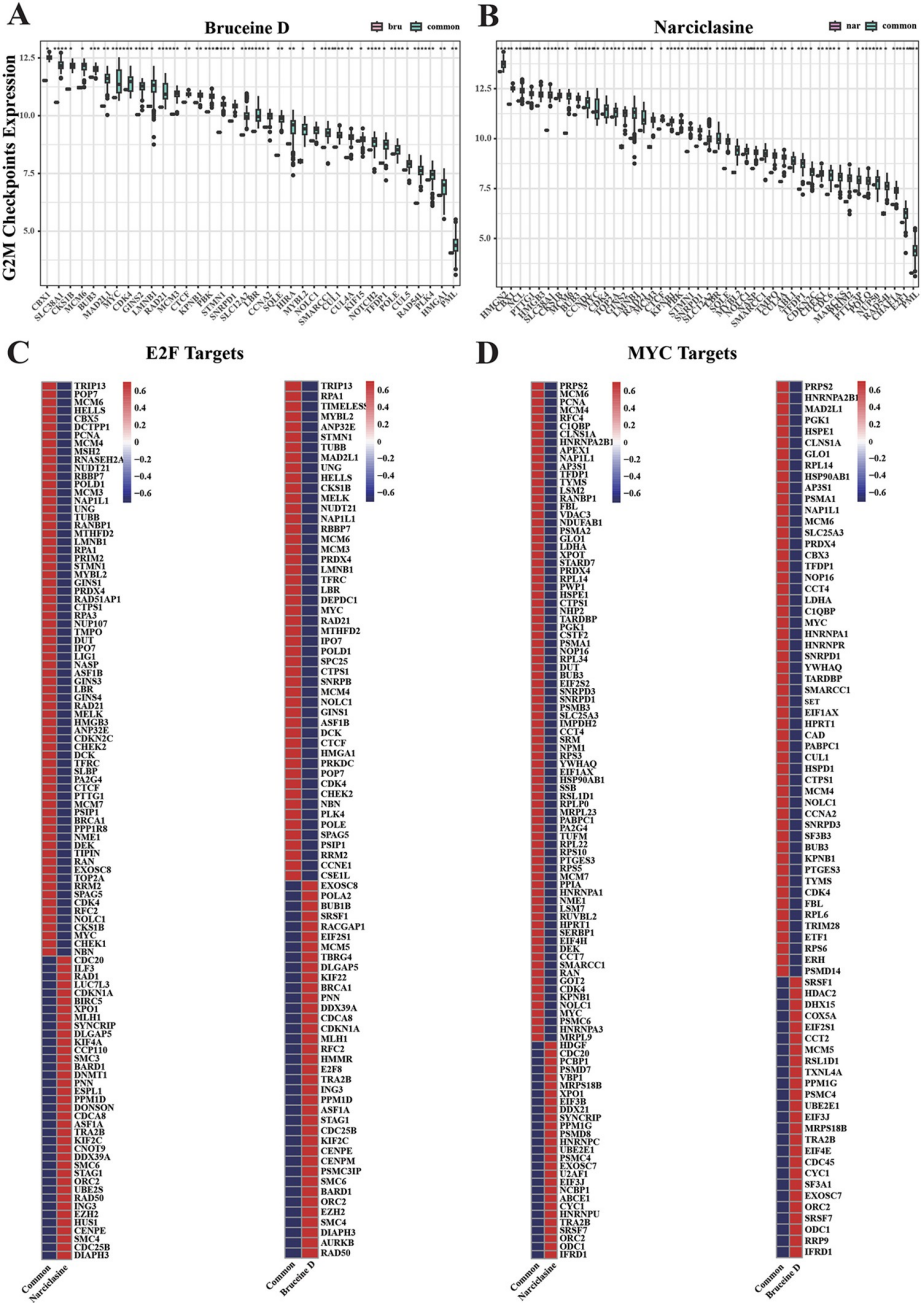
The expression of cell cycle related genes in comparing with common groups. (A) The expression of genes related to G2M checkpoints pathway in MCF7 cells treated by Bruceine D. (B) The expression of genes related to G2M checkpoints pathway in MCF7 cells treated by Narciclasine. (C) The expression of genes related to E2F targets pathway in MCF7 cells treated by Narciclasine and Bruceine D. (D) The expression of genes related to MYC targets pathway in MCF7 cells treated by Narciclasine and Bruceine D. *p < 0.05, **p < 0.01, ***p < 0.001, ****p < 0.0001. The vertical axis in Fig C and D means the expression level of genes, where the red square represents high expression, and the blue square represents low expression.

**Fig 5:**
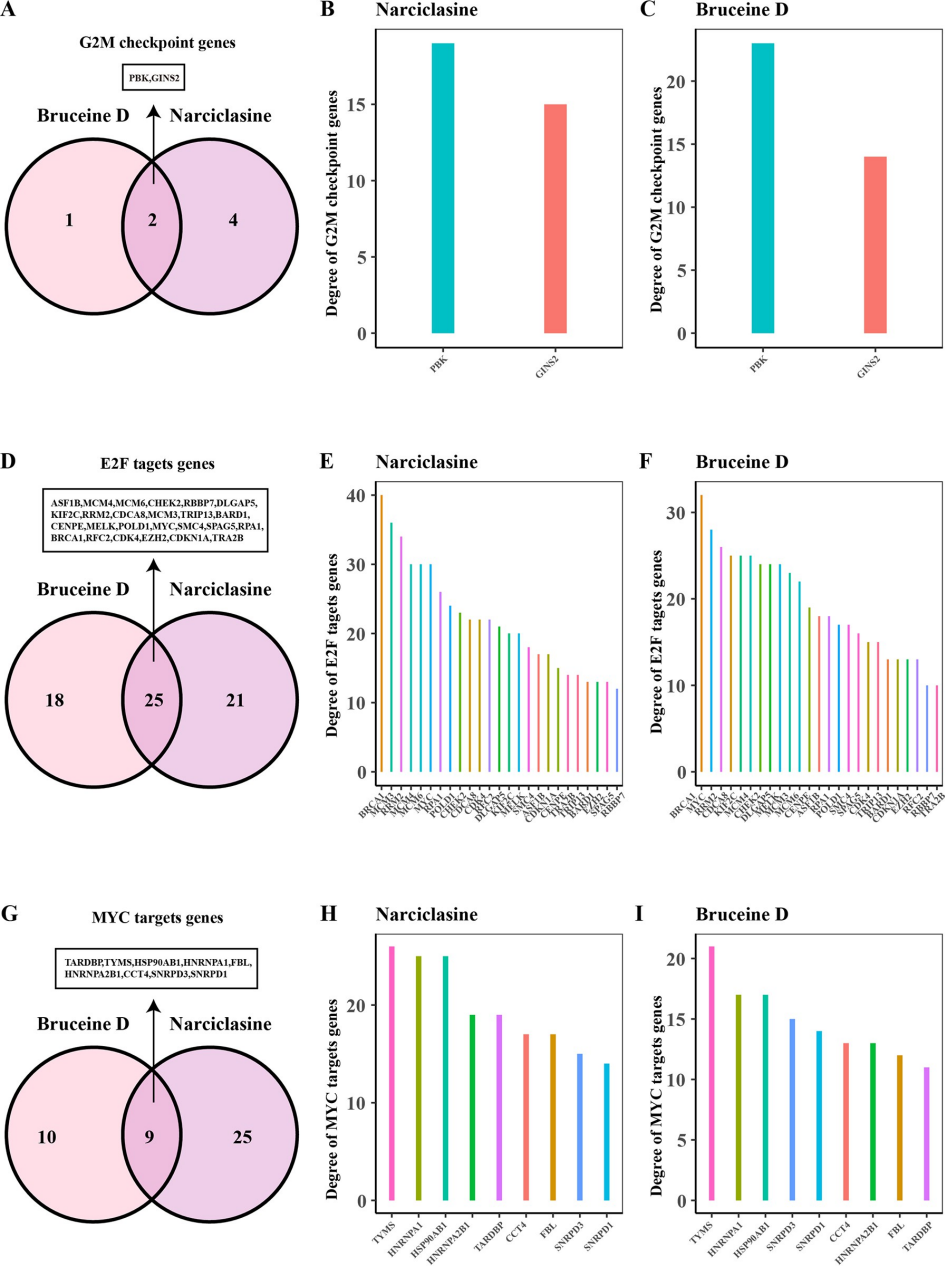
The shared genes between Narciclasine and Bruceine D treatment groups. (A) G2M checkpoints genes shared by Bruceine D and Narciclasine in MCF7 cells. (B) The connectivity degree of G2M checkpoints gene in Narciclasine treatment group. (C) The connectivity degree of G2M checkpoints gene in Bruceine D treatment group. (D) E2F target genes shared by Bruceine D and Narciclasine in MCF7 cells. (E) The connectivity degree of E2F target genes in Narciclasine treatment group. (F) The connectivity degree of E2F target genes in Bruceine D treatment group. (G) MYC target genes shared by Bruceine D and Narciclasine in MCF7 cells. (H) The connectivity degree of MYC target genes in Narciclasine treatment group. (I) The connectivity degree of MYC target genes in Narciclasine treatment group.

**Fig 6:**
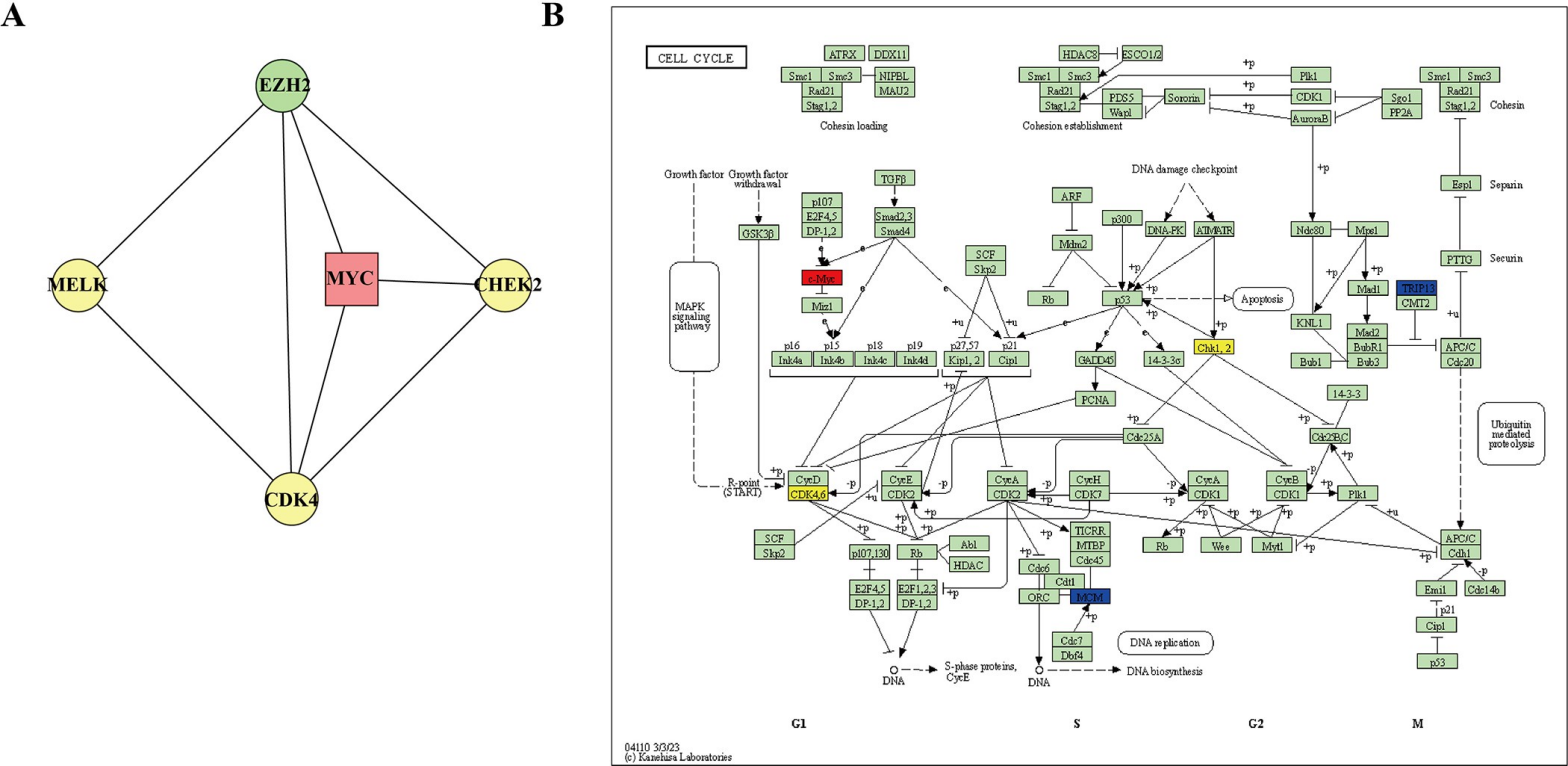
PPI analysis of 5 cell cycle related genes. (A) PPI network analysis of key cell cycle genes. (B) The position of some hub genes in cell cycle process.

Next, we investigated pathway related to cell cycle to further explore the mechanism through which Narciclasine and Bruceine D inhibited the proliferation of breast cancer cells. The expression profile in the G2M checkpoint, E2F target, MYC target v1 pathway were analyzed. Tumor cells are cells with DNA defects. Continuous activation of G2M checkpoint arrests tumor cells in G2 phase [42]. Anti-cancer strategies based on G2M checkpoints focus on targeting G2M checkpoint inactivation, forcing cancer cells to enter mitotic M phase, which ultimately leads to mitotic catastrophe and cell death [43]. The expression of E2F target genes is gradually up-regulated during G1, promoting DNA reproduction and cell division from the G1 to S stage [44]. MYC targets are a set of nucleoprotein-like oncogenes that regulate the expression of multiple genes, promote cell proliferation and metastasis, and inhibit cell apoptosis [45]. The expression of genes related to G2M checkpoint was down-regulated (**Fig 4A and 4B**), including CHEK2, MELK, CDK4. The transcription factors MYC and methyltransferase EZH2 with cell cycle regulation role in E2F targets pathway were down-regulated in the Narciclasine and Bruceine D treatment groups (**Fig 4C**). However, the genes associated with MYC target v1 pathway were not obviously involved in cell cycle regulation (**Fig 4D**). In the PPI network analysis, PBK and GINS2 were two shared genes in G2M checkpoint pathway (**Fig 5A**) between Narciclasine and Bruceine D, and the PBK had the highest connectivity degree with other genes through PPI analysis (**Fig 5B and 5C**). Narciclasine and Bruceine D in E2F target pathway had 25 shared genes including CHEK2, MELK, CDK4 (**Fig 5D**), which also possessed higher connectivity degree with other genes (**Fig 5E and 5F**). The MYC target v1 pathway had 9 shared genes (**Fig 5G**), with TYMS having the highest connectivity degree with other genes (**Fig 5H and 5I**). The high connectivity degree, the more importance of the genes and related pathways. Thus, we speculated that Narciclasine and Bruceine D could inhibit MCF7 cell proliferation through targeting MELK, CDK4, and MYC to regulate cell cycle in breast cancer. PPI analysis also disclosed a close connection between these 5 genes (**Fig 6A)**, and some of these genes were in a hub position of cell cycle process from KEGG database (**Fig 6B)**.

### The potential drug targets for Narciclasine and Bruceine D and partial validation

**Fig 7:**
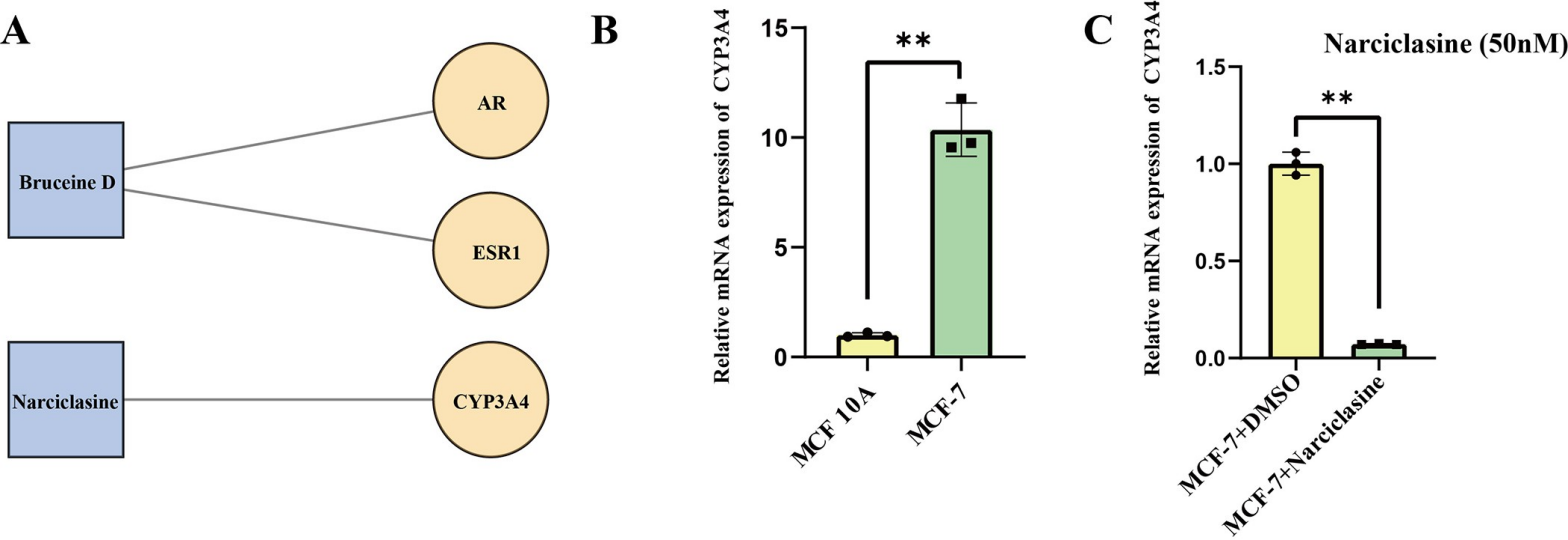
Drug target prediction and validation. (A) Predicted drug target for Narciclasine and Bruceine D. (B) The mRNA levels of CYP3A4 in normal (MCF 10A) and tumor (MCF 7) breast cells. (C) The mRNA levels of CYP3A4 in tumor (MCF 7) breast cells after Narciclasine treatment. N = 3, ** P < 0.01. The results are presented as mean ± SEM.

Drug prediction analysis uncovered that Androgen Receptor (AR) and Estrogen Receptor 1 (ESR1) were the targets for Bruceine D, and that Cytochrome P450 3A4 enzyme (CYP3A4) was the target for Narciclasine (**Fig 7A**). Next, we carried out *in vitro* experiments to confirm the connections of Narciclasine and CYP3A4. We first detected the expressions of CYP3A4 in normal and tumor breast cell lines. As shown in **Fig 7B**, the expression of CYP3A4 was up-regulated in MCF 7 cells in comparison to normal MCF 10A cells but it was significantly down-regulated when MCF 7 cells were treated with 50nM Narciclasine (**Fig 7C**). These results validated that CYP3A4 was the target for Narciclasine.

## Discussion

Increasing findings support the use of Narciclasine and Bruceine D as antitumor agents [33, 46]. Several studies have reported the antitumor mechanisms of the two drugs [47–49], but natural products often have multiple molecular targets. Therefore, based on the transcriptome data of 212 breast cell lines treated by different drugs, this study comprehensively explored the underlying antitumor targets for Narciclasine and Bruceine D.

Breast cancer is one of the most common malignant tumors to women, especially, hormone receptor-positive breast cancer [50] accounts for 60%-70% of all breast cancer cases [51] and endocrine therapy is currently the main treatment for this type of breast cancer [52]. Though treatments for the cancer have been greatly improved, a considerable number of patients will eventually develop resistance to endocrine therapy [53], leading to a poor prognosis. Previous studies indicated that endocrine resistance is resulted from multiple mechanisms, for instance, loss or mutation in estrogen receptor (ER) and deregulation of cell cycle signaling molecules [54, 55]. Thus, researchers identified novel therapeutic targets based on these mechanisms, including cyclin-dependent kinase (CDK) 4/6 inhibitors. CDK4/6 is involved in the transition from G1 to S phase, the complex cyclin D- CDK4/6 phosphorylates RB and dissociates them from the E2F to promote cell cycle progression [56]. There are various factors that can cause hyperphosphorylation of RB, for example, cyclin D is overexpressed in about half of breast cancers and this may ultimately lead to uncontrolled cell proliferation [57]. The pathway CDK-RB1-E2F targeted by CDK4/6 inhibitors is vital for inhibiting the proliferation of tumor cells and it is disrupted in a majority of cancers [58, 59]. Currently, palbociclib, ribociclib and abemaciclib as three CDK 4/6 inhibitors [60] that have remarkable effect when used in combination with endocrine therapies and they have all been approved by Food and Drug Administration (FDA) [61].

In addition, PBK is a shared down-regulated genes with the highest connectivity degrees in the Narciclasine and Bruceine D treatment groups, indicating that it played important role in breast cancer cell proliferation. PBK is a serine/threonine kinase that is considered as oncogenic protein involved in cell growth, proliferation, apoptosis and inflammation [62, 63]. PBK is aberrantly overexpressed in many cancers, such as adrenocortical carcinoma [64], hepatocellular carcinoma [65], and pancreatic cancer [66]. PBK has also been reported as a mitosis kinase activated by the complex of cyclin B1-CDK1 to promote cytokinesis through the phosphorylation of polycomb repressive complex 1(PRC1) [67]. PBK binding to PRC1 increases the phosphorylation of PRC1 at T481 site, which further elevates the phosphorylation level of cyclin B1-CDK1 to PRC1 and ultimately promotes cell cycle division [67]. Previous studies indicated that overexpression of PBK increases the cell proliferation and tumorigenesis in JB6 epidermal cells *in vivo* and *in vitro* [68, 69], while knockdown of PBK suppresses tumor growth of lung cancer cell lines [70]. Some studies also revealed that the expression levels of PBK and kinase activity are positively correlated with the G2/M phase cell numbers in prostate cancer and gastric carcinoma. Silencing or inhibiting the enzymatic activity of PBK can arrest tumor cells in G0 phase [71] because the PBK-CDK1-cyclin B complex could prolong the degradation of cyclin B [72]. Together, these findings indicates the inhibitory effect of Narciclasine and Bruceine D on cell cycle to block the tumor proliferation of breast cancer.

In this study, Narciclasine and Bruceine D showing similar pathway enrichment were screened by target prediction analysis. AR and ESR1 were the targets for Bruceine D, and CYP3A4 was the target for Narciclasine. AR is a type I nuclear receptor that regulates gene transcription for cell differentiation, proliferation, apoptosis, or angiogenesis of breast cancer [73]. The fact that AR levels exceed 70% in both primary and metastatic breast cancer suggests that AR could be a novel therapeutic target for AR+ breast cancer patients [74]. ESR1 is responsible for encoding estrogen receptor α (ER). Although targeting of ER+ breast cancer patients with endocrine therapy (ET) is currently a standard treatment [75], mutations of ESR1 is the essential driver of ET resistance [74]. Therefore, in-depth studies should be performed to probe into the effect of Bruceine D on mutated ESR1 in breast cancer. Carbon Monoxide can suppress the levels of CYP3A4 to enhance the sensitivity of human breast cancer cells to Paclitaxel [76]. In this research, we found that a high level of CYP3A4 in MCF 7 cells could be reduced by Narciclasine treatment, suggesting a new target drug for breast cancer treatment. In short, drug prediction results showed a potential therapeutic direction for breast cancer.

## Conclusion

We performed a comprehensive analysis of breast cancer cells treated by different drugs and identified several drug targets for both Narciclasine and Bruceine D. These two drugs can inhibit the growth and proliferation of breast cancer cells. The current findings are expected to contribute to the clinical development of potential therapies for breast cancer patients.

## Abbreviations

AR: Androgen Receptor
CDK4: cyclin-dependent kinase 4
CYP3A4: Cytochrome P450 3A4 enzyme
ER: Estrogen receptor α
ESR1: Estrogen Receptor 1
ET: endocrine therapyGEO, Gene Expression Omnibus
ssGSEA: single sample gene set enrichment analysis
PPI: Protein-Protein Interaction
PCA: Principal Component Analysis
MCF7: human breast cancer cell lines
MSigDB: Molecular Signatures Database
DEGs: Differentially expressed genes
SCT1: stanniocalcin-1

## References

[1] Sung H, Ferlay J, Siegel RL, Laversanne M, Soerjomataram I, Jemal A, et al. Global Cancer Statistics 2020: GLOBOCAN Estimates of Incidence and Mortality Worldwide for 36 Cancers in 185 Countries. CA: a cancer journal for clinicians. 2021;71(3):209–49. doi: 10.3322/caac.21660 33538338

[2] Shang C, Xu D. Epidemiology of Breast Cancer. Oncologie. 2022;24(4):649–63.

[3] Peintinger F. National Breast Screening Programs across Europe. Breast care (Basel, Switzerland). 2019;14(6):354–8. doi: 10.1159/000503715 31933580 PMC6940461

[4] Zhou T, Li Y, Yang L, Liu L, Ju Y, Li C. Silencing of ANXA3 expression by RNA interference inhibits the proliferation and invasion of breast cancer cells. Oncology reports. 2017;37(1):388–98. 27878264 10.3892/or.2016.5251

[5] Hong W, Dong E. The past, present and future of breast cancer research in China. Cancer letters. 2014;351(1):1–5. 24735750 10.1016/j.canlet.2014.04.007

[6] Kim JY, Jung EJ, Park HJ, Lee JH, Song EJ, Kwag SJ, et al. Tumor-Suppressing Effect of Silencing of Annexin A3 Expression in Breast Cancer. Clinical breast cancer. 2018;18(4):e713–e9. doi: 10.1016/j.clbc.2017.11.009 29217453

[7] Cortazar P, Zhang L, Untch M, Mehta K, Costantino JP, Wolmark N, et al. Pathological complete response and long-term clinical benefit in breast cancer: the CTNeoBC pooled analysis. Lancet (London, England). 2014;384(9938):164–72. doi: 10.1016/S0140-6736(13)62422-8 24529560

[8] Huang J, Bai X, Xie X, Chen L, Lan X, Zhang Q, et al. A Real-World Study on Oral Vinorelbine for the Treatment of Metastatic Breast Cancer. Oncologie. 2022;24(1):131–45.

[9] Zhang H, Wang X, Yu Y, Yang Z. Progression of Exosome-Mediated Chemotherapy Resistance in Cancer. Oncologie. 2022;24(2):247–59.

[10] Colone M, Calcabrini A, Stringaro A. Drug Delivery Systems of Natural Products in Oncology. Molecules (Basel, Switzerland). 2020;25(19). 10.3390/molecules25194560 PMC7582809 33036240

[11] Sin ZW, Bhardwaj V, Pandey AK, Garg M. A brief overview of antitumoral actions of bruceine D. Exploration of targeted anti-tumor therapy. 2020;1(4):200–17. doi: 10.37349/etat.2020.00013 36046775 PMC9400783

[12] Fürst R. Narciclasine—an Amaryllidaceae Alkaloid with Potent Antitumor and Anti-Inflammatory Properties. Planta medica. 2016;82(16):1389–94. doi: 10.1055/s-0042-115034 27542176

[13] Wenzel ES, Singh ATK. Cell-cycle Checkpoints and Aneuploidy on the Path to Cancer. In vivo (Athens, Greece). 2018;32(1):1–5. doi: 10.21873/invivo.11197 29275292 PMC5892633

[14] Icard P, Fournel L, Wu Z, Alifano M, Lincet H. Interconnection between Metabolism and Cell Cycle in Cancer. Trends in biochemical sciences. 2019;44(6):490–501. doi: 10.1016/j.tibs.2018.12.007 30655165

[15] Wang Z. Cell Cycle Progression and Synchronization: An Overview. Methods in molecular biology (Clifton, NJ). 2022;2579:3–23. doi: 10.1007/978-1-0716-2736-5_1 36045194

[16] Malumbres M, Barbacid M. Cell cycle, CDKs and cancer: a changing paradigm. Nature reviews Cancer. 2009;9(3):153–66. doi: 10.1038/nrc2602 19238148

[17] Lim S, Kaldis P. Cdks, cyclins and CKIs: roles beyond cell cycle regulation. Development (Cambridge, England). 2013;140(15):3079–93. doi: 10.1242/dev.091744 23861057

[18] Pines J. Cyclins and cyclin-dependent kinases: theme and variations. Advances in cancer research. 1995;66:181–212. doi: 10.1016/s0065-230x(08)60254-7 7793314

[19] Johnson N, Shapiro GI. Cyclin-dependent kinases (cdks) and the DNA damage response: rationale for cdk inhibitor-chemotherapy combinations as an anticancer strategy for solid tumors. Expert opinion on therapeutic targets. 2010;14(11):1199–212. doi: 10.1517/14728222.2010.525221 20932174 PMC3957489

[20] Mandigo AC, Tomlins SA, Kelly WK, Knudsen KE. Relevance of pRB Loss in Human Malignancies. Clinical cancer research: an official journal of the American Association for Cancer Research. 2022;28(2):255–64. doi: 10.1158/1078-0432.CCR-21-1565 34407969 PMC9306333

[21] Kassab A, Gupta I, Moustafa AA. Role of E2F transcription factor in oral cancer: Recent insight and advancements. Seminars in cancer biology. 2023;92:28–41. doi: 10.1016/j.semcancer.2023.03.004 36924812

[22] Pandey K, An HJ, Kim SK, Lee SA, Kim S, Lim SM, et al. Molecular mechanisms of resistance to CDK4/6 inhibitors in breast cancer: A review. International journal of cancer. 2019;145(5):1179–88. 30478914 10.1002/ijc.32020 PMC6767051

[23] Dong SH, Liu J, Ge YZ, Dong L, Xu CH, Ding J, et al. Chemical constituents from Brucea javanica. Phytochemistry. 2013;85:175–84. 23009875 10.1016/j.phytochem.2012.08.018

[24] Huang R, Zhang L, Jin J, Zhou Y, Zhang H, Lv C, et al. Bruceine D inhibits HIF-1α-mediated glucose metabolism in hepatocellular carcinoma by blocking ICAT/β-catenin interaction. Acta pharmaceutica Sinica B. 2021;11(11):3481–92. 34900531 10.1016/j.apsb.2021.05.009 PMC8642446

[25] Fan J, Ren D, Wang J, Liu X, Zhang H, Wu M, et al. Bruceine D induces lung cancer cell apoptosis and autophagy via the ROS/MAPK signaling pathway in vitro and in vivo. Cell death & disease. 2020;11(2):126. 32071301 10.1038/s41419-020-2317-3 PMC7028916

[26] Qiu Y, Fang B, Thuy NTT, Li A, Yoo HM, Zheng X, et al. Narciclasine suppresses esophageal cancer cell proliferation and migration by inhibiting the FAK signaling pathway. European journal of pharmacology. 2022;921:174669. 35248554 10.1016/j.ejphar.2021.174669

[27] Mei J, Wang T, Zhao S, Zhang Y. Osthole Inhibits Breast Cancer Progression through Upregulating Tumor Suppressor GNG7. Journal of oncology. 2021;2021:6610511. 33727922 10.1155/2021/6610511 PMC7937475

[28] Davis S, Meltzer PS. GEOquery: a bridge between the Gene Expression Omnibus (GEO) and BioConductor. Bioinformatics (Oxford, England). 2007;23(14):1846–7. doi: 10.1093/bioinformatics/btm254 17496320

[29] Liberzon A, Birger C, Thorvaldsdóttir H, Ghandi M, Mesirov JP, Tamayo P. The Molecular Signatures Database (MSigDB) hallmark gene set collection. Cell systems. 2015;1(6):417–25. 26771021 10.1016/j.cels.2015.12.004 PMC4707969

[30] Hänzelmann S, Castelo R, Guinney J. GSVA: gene set variation analysis for microarray and RNA-seq data. BMC bioinformatics. 2013;14:7. doi: 10.1186/1471-2105-14-7 23323831 PMC3618321

[31] Tang Y, Li M, Wang J, Pan Y, Wu FX. CytoNCA: a cytoscape plugin for centrality analysis and evaluation of protein interaction networks. Bio Systems. 2015;127:67–72. doi: 10.1016/j.biosystems.2014.11.005 25451770

[32] Fang S, Dong L, Liu L, Guo J, Zhao L, Zhang J, et al. HERB: a high-throughput experiment- and reference-guided database of traditional Chinese medicine. Nucleic acids research. 2021;49(D1):D1197–d206. 33264402 10.1093/nar/gkaa1063 PMC7779036

[33] Lv C, Huang Y, Huang R, Wang Q, Zhang H, Jin J, et al. Narciclasine targets STAT3 via distinct mechanisms in tamoxifen-resistant breast cancer cells. Mol Ther Oncolytics. 2022;24:340–54. doi: 10.1016/j.omto.2021.12.025 35118192 PMC8783118

[34] Li W, Xu M, Li Y, Huang Z, Zhou J, Zhao Q, et al. Comprehensive analysis of the association between tumor glycolysis and immune/inflammation function in breast cancer. Journal of translational medicine. 2020;18(1):92. doi: 10.1186/s12967-020-02267-2 32070368 PMC7029444

[35] Griffioen AW, Dudley AC. Angiogenesis: a year in review. Angiogenesis. 2021;24(2):195–6. doi: 10.1007/s10456-021-09798-2 34050879 PMC8164393

[36] Deng F, Zhou R, Lin C, Yang S, Wang H, Li W, et al. Tumor-secreted dickkopf2 accelerates aerobic glycolysis and promotes angiogenesis in colorectal cancer. Theranostics. 2019;9(4):1001–14. doi: 10.7150/thno.30056 30867812 PMC6401398

[37] Chang AC, Doherty J, Huschtscha LI, Redvers R, Restall C, Reddel RR, et al. STC1 expression is associated with tumor growth and metastasis in breast cancer. Clinical & experimental metastasis. 2015;32(1):15–27. doi: 10.1007/s10585-014-9687-9 25391215

[38] Dai D, Wang Q, Li X, Liu J, Ma X, Xu W. Klotho inhibits human follicular thyroid cancer cell growth and promotes apoptosis through regulation of the expression of stanniocalcin-1. Oncology reports. 2016;35(1):552–8. doi: 10.3892/or.2015.4358 26531219

[39] Liu G, Yang G, Chang B, Mercado-Uribe I, Huang M, Zheng J, et al. Stanniocalcin 1 and ovarian tumorigenesis. Journal of the National Cancer Institute. 2010;102(11):812–27. doi: 10.1093/jnci/djq127 20484106 PMC2879417

[40] Ma X, Gu L, Li H, Gao Y, Li X, Shen D, et al. Hypoxia-induced overexpression of stanniocalcin-1 is associated with the metastasis of early stage clear cell renal cell carcinoma. Journal of translational medicine. 2015;13:56. doi: 10.1186/s12967-015-0421-4 25740019 PMC4337255

[41] Du YZ, Gu XH, Cheng SF, Li L, Liu H, Hu LP, et al. The oncogenetic role of stanniocalcin 1 in lung adenocarcinoma: a promising serum candidate biomarker for tracking lung adenocarcinoma progression. Tumour biology: the journal of the International Society for Oncodevelopmental Biology and Medicine. 2016;37(4):5633–44. doi: 10.1007/s13277-015-4431-x 26577859

[42] Barnaba N, LaRocque JR. Targeting cell cycle regulation via the G2-M checkpoint for synthetic lethality in melanoma. Cell cycle (Georgetown, Tex). 2021;20(11):1041–51. doi: 10.1080/15384101.2021.1922806 33966611 PMC8208119

[43] Vitale I, Galluzzi L, Castedo M, Kroemer G. Mitotic catastrophe: a mechanism for avoiding genomic instability. Nature reviews Molecular cell biology. 2011;12(6):385–92. doi: 10.1038/nrm3115 21527953

[44] Kent LN, Leone G. The broken cycle: E2F dysfunction in cancer. Nature reviews Cancer. 2019;19(6):326–38. 31053804 10.1038/s41568-019-0143-7

[45] Dang CV. MYC on the path to cancer. Cell. 2012;149(1):22–35. doi: 10.1016/j.cell.2012.03.003 22464321 PMC3345192

[46] Cipriani C, Pacheco MP, Kishk A, Wachich M, Abankwa D, Schaffner-Reckinger E, et al. Bruceine D Identified as a Drug Candidate against Breast Cancer by a Novel Drug Selection Pipeline and Cell Viability Assay. Pharmaceuticals (Basel). 2022;15(2). doi: 10.3390/ph15020179 35215292 PMC8875459

[47] TangW, HuY, TuK, GongZ, ZhuM, YangT, et al. Targeting Trop2 by Bruceine D suppresses breast cancer metastasis by blocking Trop2/β-catenin positive feedback loop. J Adv Res. 2023.10.1016/j.jare.2023.05.01237271476

[48] Tian S, Jing R, Zhang W. Network-Based Approach to Identify the Antiproliferative Mechanisms of Bruceine D in Breast Cancer From the Cancer Genome Atlas. Front Oncol. 2020;10:1001. doi: 10.3389/fonc.2020.01001 32714860 PMC7343963

[49] Liao M, Qin R, Huang W, Zhu HP, Peng F, Han B, et al. Targeting regulated cell death (RCD) with small-molecule compounds in triple-negative breast cancer: a revisited perspective from molecular mechanisms to targeted therapies. J Hematol Oncol. 2022;15(1):44. doi: 10.1186/s13045-022-01260-0 35414025 PMC9006445

[50] Szklarczyk D, Kirsch R, Koutrouli M, Nastou K, Mehryary F, Hachilif R, et al. The STRING database in 2023: protein-protein association networks and functional enrichment analyses for any sequenced genome of interest. Nucleic acids research. 2023;51(D1):D638–d46. doi: 10.1093/nar/gkac1000 36370105 PMC9825434

[51] Hart CD, Migliaccio I, Malorni L, Guarducci C, Biganzoli L, Di Leo A. Challenges in the management of advanced, ER-positive, HER2-negative breast cancer. Nature reviews Clinical oncology. 2015;12(9):541–52. doi: 10.1038/nrclinonc.2015.99 26011489

[52] Flaum LE, Gradishar WJ. Advances in Endocrine Therapy for Postmenopausal Metastatic Breast Cancer. Cancer treatment and research. 2018;173:141–54. doi: 10.1007/978-3-319-70197-4_9 29349762

[53] Ouifki R, Oke SI. Mathematical model for the estrogen paradox in breast cancer treatment. Journal of mathematical biology. 2022;84(4):28. doi: 10.1007/s00285-022-01729-z 35239041

[54] Hanker AB, Sudhan DR, Arteaga CL. Overcoming Endocrine Resistance in Breast Cancer. Cancer cell. 2020;37(4):496–513. doi: 10.1016/j.ccell.2020.03.009 32289273 PMC7169993

[55] Zhu D, Yang J, Xu J. G-Protein-Coupled Estrogen Receptor Enhances the Stemness of Triple-Negative Breast Cancer Cells and Promotes Malignant Characteristics. Oncologie. 2022;24(3):471–82.

[56] Clark AS, Karasic TB, DeMichele A, Vaughn DJ, O’Hara M, Perini R, et al. Palbociclib (PD0332991)-a Selective and Potent Cyclin-Dependent Kinase Inhibitor: A Review of Pharmacodynamics and Clinical Development. JAMA oncology. 2016;2(2):253–60. doi: 10.1001/jamaoncol.2015.4701 26633733

[57] Hamilton E, Infante JR. Targeting CDK4/6 in patients with cancer. Cancer treatment reviews. 2016;45:129–38. doi: 10.1016/j.ctrv.2016.03.002 27017286

[58] Klein ME, Kovatcheva M, Davis LE, Tap WD, Koff A. CDK4/6 Inhibitors: The Mechanism of Action May Not Be as Simple as Once Thought. Cancer cell. 2018;34(1):9–20. doi: 10.1016/j.ccell.2018.03.023 29731395 PMC6039233

[59] Goel S, DeCristo MJ, McAllister SS, Zhao JJ. CDK4/6 Inhibition in Cancer: Beyond Cell Cycle Arrest. Trends in cell biology. 2018;28(11):911–25. doi: 10.1016/j.tcb.2018.07.002 30061045 PMC6689321

[60] Braal CL, Jongbloed EM, Wilting SM, Mathijssen RHJ, Koolen SLW, Jager A. Inhibiting CDK4/6 in Breast Cancer with Palbociclib, Ribociclib, and Abemaciclib: Similarities and Differences. Drugs. 2021;81(3):317–31. doi: 10.1007/s40265-020-01461-2 33369721 PMC7952354

[61] Corona SP, Generali D. Abemaciclib: a CDK4/6 inhibitor for the treatment of HR+/HER2- advanced breast cancer. Drug design, development and therapy. 2018;12:321–30. doi: 10.2147/DDDT.S137783 29497278 PMC5818877

[62] Zhao R, Choi BY, Wei L, Fredimoses M, Yin F, Fu X, et al. Acetylshikonin suppressed growth of colorectal tumour tissue and cells by inhibiting the intracellular kinase, T-lymphokine-activated killer cell-originated protein kinase. British journal of pharmacology. 2020;177(10):2303–19. doi: 10.1111/bph.14981 31985814 PMC7174886

[63] Zhao R, Huang H, Choi BY, Liu X, Zhang M, Zhou S, et al. Cell growth inhibition by 3-deoxysappanchalcone is mediated by directly targeting the TOPK signaling pathway in colon cancer. Phytomedicine: international journal of phytotherapy and phytopharmacology. 2019;61:152813. doi: 10.1016/j.phymed.2018.12.036 31035049

[64] Kar A, Zhang Y, Yacob BW, Saeed J, Tompkins KD, Bagby SM, et al. Targeting PDZ-binding kinase is anti-tumorigenic in novel preclinical models of ACC. Endocrine-related cancer. 2019;26(10):765–78. doi: 10.1530/ERC-19-0262 31325906 PMC6938568

[65] Yang QX, Zhong S, He L, Jia XJ, Tang H, Cheng ST, et al. PBK overexpression promotes metastasis of hepatocellular carcinoma via activating ETV4-uPAR signaling pathway. Cancer letters. 2019;452:90–102. doi: 10.1016/j.canlet.2019.03.028 30914208

[66] Hinzman CP, Aljehane L, Brown-Clay JD, Kallakury B, Sonahara F, Goel A, et al. Aberrant expression of PDZ-binding kinase/T-LAK cell-originated protein kinase modulates the invasive ability of human pancreatic cancer cells via the stabilization of oncoprotein c-MYC. Carcinogenesis. 2018;39(12):1548–59. doi: 10.1093/carcin/bgy114 30165468

[67] Abe Y, Takeuchi T, Kagawa-Miki L, Ueda N, Shigemoto K, Yasukawa M, et al. A mitotic kinase TOPK enhances Cdk1/cyclin B1-dependent phosphorylation of PRC1 and promotes cytokinesis. Journal of molecular biology. 2007;370(2):231–45. doi: 10.1016/j.jmb.2007.04.067 17512944

[68] Yang YF, Pan YH, Cao Y, Fu J, Yang X, Zhang MF, et al. PDZ binding kinase, regulated by FoxM1, enhances malignant phenotype via activation of β-Catenin signaling in hepatocellular carcinoma. Oncotarget. 2017;8(29):47195–205. 28525379 10.18632/oncotarget.17587 PMC5564556

[69] Fan X, Tao J, Cai Z, Fredimoses M, Wu J, Jiang Z, et al. Eupafolin Suppresses Esophagus Cancer Growth by Targeting T-LAK Cell-Originated Protein Kinase. Frontiers in pharmacology. 2019;10:1248. doi: 10.3389/fphar.2019.01248 31708778 PMC6822407

[70] Wei DC, Yeh YC, Hung JJ, Chou TY, Wu YC, Lu PJ, et al. Overexpression of T-LAK cell-originated protein kinase predicts poor prognosis in patients with stage I lung adenocarcinoma. Cancer science. 2012;103(4):731–8. doi: 10.1111/j.1349-7006.2011.02197.x 22192142 PMC7659243

[71] Ohashi T, Komatsu S, Ichikawa D, Miyamae M, Okajima W, Imamura T, et al. Overexpression of PBK/TOPK Contributes to Tumor Development and Poor Outcome of Esophageal Squamous Cell Carcinoma. Anticancer research. 2016;36(12):6457–66. 27919968 10.21873/anticanres.11244

[72] Huang H, Lee MH, Liu K, Dong Z, Ryoo Z, Kim MO. PBK/TOPK: An Effective Drug Target with Diverse Therapeutic Potential. Cancers. 2021;13(9). 10.3390/cancers13092232 PMC8124186 34066486

[73] Venema CM, Bense RD, Steenbruggen TG, Nienhuis HH, Qiu SQ, van Kruchten M, et al. Consideration of breast cancer subtype in targeting the androgen receptor. Pharmacol Ther. 2019;200:135–47. 31077689 10.1016/j.pharmthera.2019.05.005

[74] Anestis A, Zoi I, Papavassiliou AG, Karamouzis MV. Androgen Receptor in Breast Cancer-Clinical and Preclinical Research Insights. Molecules (Basel, Switzerland). 2020;25(2). doi: 10.3390/molecules25020358 31952272 PMC7024330

[75] Loibl S, Poortmans P, Morrow M, Denkert C, Curigliano G. Breast cancer. Lancet (London, England). 2021;397(10286):1750–69. doi: 10.1016/S0140-6736(20)32381-3 33812473

[76] Kawahara B, Faull KF, Janzen C, Mascharak PK. Carbon Monoxide Inhibits Cytochrome P450 Enzymes CYP3A4/2C8 in Human Breast Cancer Cells, Increasing Sensitivity to Paclitaxel. J Med Chem. 2021;64(12):8437–46. doi: 10.1021/acs.jmedchem.1c00404 34097831

